# Risk factors for the development of aspiration pneumonia in elderly patients with femoral neck and trochanteric fractures: A retrospective study of a patient cohort

**DOI:** 10.1097/MD.0000000000019108

**Published:** 2020-02-14

**Authors:** Toshihiro Higashikawa, Kenji Shigemoto, Kenichi Goshima, Daisuke Usuda, Masashi Okuro, Manabu Moriyama, Hiromi Inujima, Masahiro Hangyou, Kimiko Usuda, Shigeto Morimoto, Tadami Matsumoto, Shigeki Takashima, Tsugiyasu Kanda, Takeshi Sawaguchi

**Affiliations:** a Department of Geriatric Medicine, Kanazawa Medical University Himi Municipal Hospital, 1130, Kurakawa, Himi, Toyama 935-8531; b Kanazawa Medical University, Uchinada, Kahoku-gun, Ishikawa 920-0293; c Department of Orthopedics and Joint Reconstructive Surgery, Toyama Municipal Hospital, Hokubumachi, Imaizumi, Toyama 939-8511; d Department of Geriatric Medicine, Kanazawa Medical University, Uchinada, Kahoku-gun, Ishikawa 920-0293; e Department of Urology, Kanazawa Medical University Himi Municipal Hospital, Kurakawa, Himi, Toyama 935-8531; f Toyama Municipal Hospital, Hokubumachi, Imaizumi, Toyama, Toyama 939-8511; g Department of Orthopedic Medicine, Kanazawa Medical University, Uchinada, Kahoku-gun, Ishikawa 920-0293, Japan.

**Keywords:** albumin, aspiration pneumonia, elderly, hip fractures

## Abstract

Aspiration pneumonia (AP) has been recognized as one of the most common postoperative complications after hip surgery in elderly. The objective of the present study was to evaluate risk for postoperative complications of AP in elderly patients with femoral neck fractures.

We recruited 426 patients (age 84.9 ± 7.4 years) with a history of hip surgery carried out at Toyama Municipal Hospital. AP occurred in 18 out of 426 cases (4.23%). Statistical test has found significant differences in age, gender, serum albumin level, and cognitive impairment, between AP and non-AP groups. Subsequently multiple logistic regression analysis was conducted to investigate the risk factors for AP, including age, gender, serum albumin, cognitive impairment, and activities of daily living (ADL). Adjusted odds ratio showed significant differences in age, gender, and serum albumin, whereas no significant differences were found in cognitive impairment and ADL.

This study suggested that serum albumin seemed to be a risk factor for AP but were necessary to assess under adjustment of confounding factors, including age and gender. Monitoring serum albumin level seemed to be important for the postoperative management of AP, especially in elderly patients receiving surgery of femoral neck and trochanteric fractures.

## 1 Introduction

The incidence of hip fracture in the elderly has been increased rapidly from 1.7 million in 1990 to an estimated 6.3 million in 2050 worldwide.^[1]^ Approximately one third of these patients die within the first year.^[2]^ A nationwide survey of hip fractures by the Japanese Orthopaedic Association (JOA) from 1998 to 2008 found a drastic increase in incidence.^[3]^ In consequence, collaboration between geriatrician and orthopedist has been conducted in various ways in Japan. Such collaboration has been conducted in Toyama city, which is located in the middle district in Toyama Prefecture, which has an area of approximately 1200 km and has about 400,000 residents.^[4]^ The proportion of elderly people aged 65 and above, to all the residents in Toyama city is over 30%. Toyama Municipal Hospital is a core hospital in Toyama Prefecture, which has approximately 600 beds. In the hospital, an Elderly Bone Femoral Neck and Trochanteric Fracture Center has been installed with advanced technology and dedicated staffs to conduct surgery in patients with acute phase femoral neck and trochanteric fracture. Co-management of hip fracture patients by orthopedic surgeons and geriatricians is suggested to be effective to reduce length of hospital stay without negatively affecting major patient outcomes.^[2]^ On this account, multidisciplinary cooperation could be effective as conducted in Toyama Municipal Hospital.

The cause of mortality in elderly people with hip fractures mostly attributed to postoperative complications, such as wound infections, pneumonia, deep venous thromboses, respiratory and urinary infections, and cardiovascular events.^[5–12]^ The consequences of hip fractures in elderly persons also include a deterioration in functional capacity to perform activities that enable independent living.^[13,14]^

Among these patients, cases of complicated pneumonia are frequently encountered, but there are few reports investigated complications of pneumonia.^[14,15]^ The majority of postoperative pneumonia cases are caused by gram-negative, aerobic bacteria including *Pseudomonas*, *Klebsiella*, and *Enterobacter* species, among others. With regard to gram-positive bacteria, methicillin-resistant *Staphyloccocus aureus* is the most common cause. Even more troubling is the growing resistance to antimicrobial medications, thereby making intervention and treatment increasingly more difficult.^[8]^ The hyperglycemia and lower platelet count are also known as causes for postoperative pneumonia after hip surgery.^[6]^

Therefore, the present study was retrospectively investigated on the cases suffering from aspiration pneumonia (AP), which were hospitalized for femoral fracture and received operation and rehabilitation. In addition, we examined risk factors for pneumonia, particularly focusing on risk factors for AP to identify some measures to raise the patient's QOL.

## 2 Materials and methods

This retrospective study was carried out under approval (2018-06) of the Clinical Research Ethics Committee of Toyama Municipal Hospital.

Demographic data were collected by physicians, specialist nurses, pharmacists, and medical affairs. The existence of the AP was also examined in the same division.

The AP was diagnosed by identifying dorsal inferior pulmonary infiltrates in postoperative chest by CT scan. The cases were defined as positive of AP (AP positive) and others were defined as negative of AP (AP negative). Definition of AP was made based on the Japanese Respiratory Society (JRS) guidelines for the management of hospital-acquired pneumonia, namely overt aspiration (apparent aspiration), a condition in which aspiration was strongly expected, or the existence of abnormal swallowing function or dysphagia^[16]^ as well as the characteristics of the surgical and anesthetic act.^[17]^ In the present study, the diagnosis of dysphagia was conducted in all cases by three-ounce water swallow test.^[18]^ In parallel, physicians checked history of dysphagia, stroke, and existence of cough reflex for all cases. Serum albumin concentration was measured and the value itself was used for the following statistical analysis as continuous variables. In addition, the data were divided into high albumin (≥3.5 g/dL) and low albumin (<3.5 g/dL) groups that were used for the following analysis as dichotomous variables.

Cognitive disorders were diagnosed by neuropsychiatrists. Degree of independence in everyday life on each case was assessed by a conventional activities of daily living (ADL) scale, which is classified by the four groups (J, A, B, C). The group J is mostly assistance-free, the group A is semi-bedridden mostly active during daytime, the group B is bedridden requiring assistance for daily activities, and the group C is bedridden requires assistance in bed. We divided them into two groups, J A (assistance non-required), and B C (assistance-required) group.

Data were collected by reviewing electronic medical records held at our hospital.

The present study consists of continuous data, such as age, serum albumin concentration, which were statistically evaluated by mean, standard deviation, and Student *t* test.

The present study also consists of dichotomous data, such as gender, categorized serum albumin concentration, cognitive disorder, ADL, and history and the number of diseases, which were statistically evaluated by Fisher exact test.

Logistic regression analyses were performed using dichotomous AP positive and AP negative as dependent variables, and age, gender, serum albumin, cognitive disorder, and ADL as independent variables.

All statistical analyses were two-tailed, *P* < .05 regarded as significant, including Student *t* test, chi-square test, and logistic regression analyses performed using EZR (Saitama Medical Center, Jichi Medical University, Saitama, Japan).^[19]^

## 3 Results

**Figure 1:**
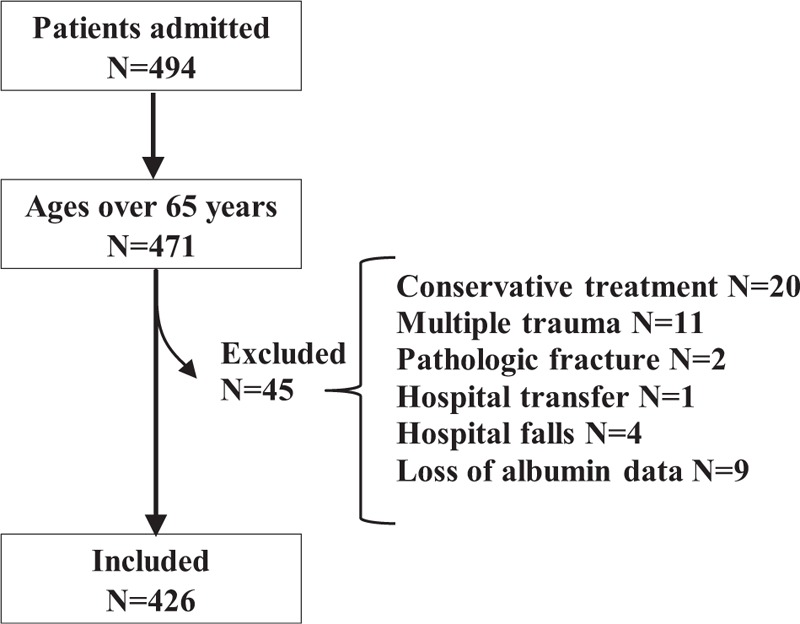
Flow chart of the study.

Initially 494 patients were admitted to the Toyama Municipal Hospital from January 1, 2016 to December 31, 2018, excluding 23 patients younger than 65 years, 20 patients with conservative treatment, 11 patients with multiple trauma, two patients with pathologic fracture, one patient with hospital transfer, four patients with in-hospital falls, and nine patients with no measurement of serum albumin. Finally, 426 patients were included and used as an analysis set of the present study as depicted in Figure 1.

Among the 426 cases, 18 cases showed symptoms of increased postprandial sputum, moist itching, decreased SpO_2_, and bulging on auscultation more than 3 days after surgery. The three-ounce water swallow test was conducted in these 18 cases, in which four cases showed mild dysphasia, 12 cases showed moderate dysphasia, and two cases showed severe dysphasia. Subsequently, CT images were taken in the cases, which showed a significant infiltrative shadow in the lower lung. Based on these results, the 18 cases were diagnosed as AP with high confidence.

Table 1 shows patient characteristics at the enrollment in this study. The patient population (N = 426) were divided into AP negative (N = 408) and AP positive (N = 18) groups. The circulatory disease includes chronic heart failure, arrhythmias, coronary artery disease, valvular disease (surgery), and aneurysm.

**Table 1:**
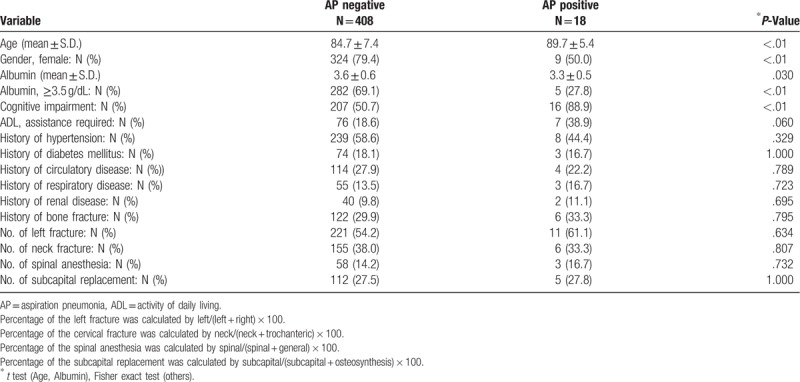
Patient characterictics at baseline.

The respiratory disease includes chronic obstructive disease, bronchitis, asthma, tuberculosis, pneumonia, pulmonary infarction, and lung cancers.

The renal disease includes patients with renal failure (acute and chronic), pyelonephritis, urinary calculus, and renal cancer.

Table 1 shows that age, gender, albumin, and cognitive impairment showed significant differences whereas ADL, history of hypertension, history of diabetes mellitus, history of circulatory disease, history of respiratory disease, history of renal disease, history of bone fracture, number of left fracture, number of neck fracture, number of spinal anesthesia, and the number of subcapital replacement showed no significant differences between the groups.

Table 2 shows the univariate and multiple logistic regression analysis. Univariate analysis showed that all variables were statistically significant, associated with the occurrence of AP. On the other hand, multivariate analysis showed that age, gender, and categorized serum albumin showed significant differences while no significances were found in cognitive impairment and ADL associated with the occurrence of AP.

**Table 2:**
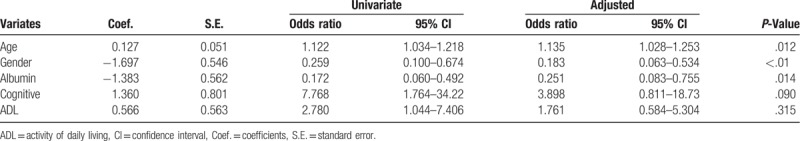
Results of logistic regression analysis.

**Figure 2:**
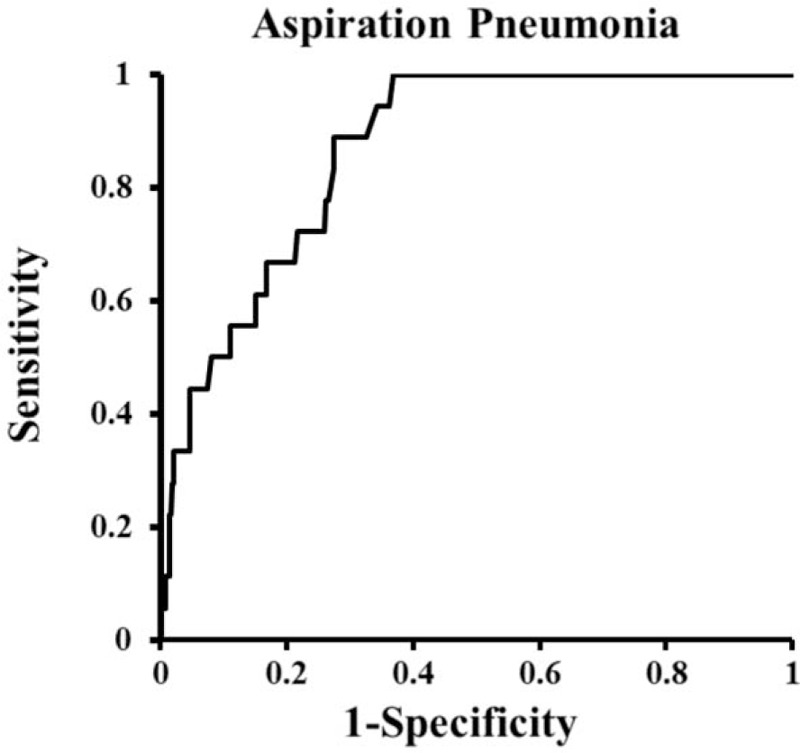
ROC curves of the logistic regression model. ROC = receiver operating characteristic.

Figure 2 shows the ROC curve of the logistic models, AP positive and AP negative as dichotomous dependent variables, and age, gender, albumin, cognitive disorder, and ADL as independent variables. The area under the curve and its 95% confidence intervals are as follows: 0.867 (0.805–0.928).

## 4 Discussion

Hip fracture is a common cause of long hospital stay in the elderly. In the Japanese practice guideline for treatment of hip fractures suggested that pneumonia is the most common postoperative complication of hip fractures, with an incidence of 3.2%, and the pneumonia is a most common complication of death during hospitalization, accounting for 30% to 44%.^[20]^ Regarding the risk factors for AP that develops in association with hip fractures, a retrospective study suggested that blood hemoglobin, serum total protein level and serum albumin level were factors that were highly associated with AP.^[15]^ Our previous study also showed common risk factors relevant to urinary retention in patients with hip fractures.^[21]^ Another retrospective study also suggested that longer duration of surgery, delayed surgery, age, low body mass index and malnutrition were identified as risk factors for AP.^[22]^ Postoperative albumin drops were known to be strongly correlated with CRP increase but could already be measured 4 to 6 hours after surgery; albumin response was further related to clinical outcomes.^[23]^ These results, together with the present result have suggested that serum albumin can play a role in the pathogenesis of AP in patients with postsurgical hip fracture.

The hip fracture surgery is also known to influence on surgical site infection,^[24]^ postoperative fever^[25]^ and mortality.^[26]^ Although our study showed 18 cases in 426 total cases, that is, approximately 4%, previous studies of hip fractures caused by falls have shown rates of complications of pneumonia before and after surgery ranging from 7% to 9%.^[10–12]^ Most evidence guiding perioperative medical risk management of patients undergoing hip fracture repair focuses on cardiac and thromboembolic risk,^[9]^ but the present study suggests AP as one of the important complications for elderly patients after hip fracture repair.

This retrospective study found that serum albumin and cognitive impairment could be risk factors for AP in patients receiving post-operative surgery of femoral neck and trochanteric fractures.

The 18 AP cases showed a significant infiltrative shadow in the lower lung. It is well-known that the distribution of AP is characterized by gravity dependence showing shadow in the lower lung,^[27]^ indicating that the 18 cases we examined were AP with high confidence.

Regarding the patient background, as shown in Table 1, age, gender, serum albumin, categorized serum albumin, and cognitive impairment showed significant differences between the AP positive and AP negative groups. The results suggested that age, gender, serum albumin, and cognitive impairment were associated with AP occurrence. On the other hand, no significant differences were observed in the ADL, history of hypertension, history of diabetes mellitus, history of circulatory disease, history of respiratory disease, history of renal disease, and history of bone fracture, indicating that the AP occurrence is less associated with history of such diseases.

The results of the multiple logistic regression analyses were shown in Table 2, which suggested that albumin showed significant association with the occurrence of AP, whereas cognitive impairment and ADL were not associated with the occurrence of AP under adjustment of age and gender as covariates using appropriate logistic model (ROC_AUC = 0.867).

The present study showed association of serum albumin level with occurrence with AP, under adjustment for confounding factors, including age and gender. A previous study has also suggested significant reduction in serum albumin in AP cases with lower BMI, indicating that patients with lower BMI, including lower nutrition were vulnerable to infection.^[15]^ Therefore, monitoring the serum albumin concentration could be beneficial for prevention of AP in future.

Complications of pneumonia do not necessarily directly lead to death, but they are anticipated to result in decreased motor function and other physical activity. Aspiration pneumonitis associated with cerebrovascular accident has been reported to result in a marked decrease in Barthel Index and Modified Ranking scale.^[28]^

The limitation of this study was to assess the risk factors of pneumonia with a small sample size. The role of serum albumin on pneumonia, relevance to cognitive impairment, and its pathological mechanism remains unknown. Although the present study is a retrospective research, it could provide insight with respect to AP as a risk factor in patients with hip fracture. Future investigations into the association between AP and physical activity will be expected.

## 5 Conclusion

In conclusion, we retrospectively evaluated the risk factors for AP in patients with femoral neck and trochanteric fractures, showed that serum albumin seemed to be the risk factors for AP under adjustment for confounding factors, including age and gender. Assistance of cognitive function also likely to be important for the postoperative management of AP, specifically in elderly patients receiving surgery of femoral neck and trochanteric fractures.

## Author contributions

**Data curation:** Toshihiro Higashikawa, Kenji Shigemoto.

**Formal analysis:** Toshihiro Higashikawa, Kenichi Goshima, Hiromi Inujima.

**Funding acquisition:** Toshihiro Higashikawa.

**Investigation:** Toshihiro Higashikawa, Kenji Shigemoto, Daisuke Usuda.

**Methodology:** Toshihiro Higashikawa, Kenichi Goshima, Daisuke Usuda, Masahiro Hangyou.

**Project administration:** Toshihiro Higashikawa.

**Resources:** Toshihiro Higashikawa.

**Software:** Toshihiro Higashikawa.

**Supervision:** Masashi Okuro, Manabu Moriyama, Kimiko Usuda, Shigeto Morimoto, Tadami Matsumoto, Shigeki Takashima, Tsugiyasu Kanda, Takeshi Sawaguchi.

**Validation:** Toshihiro Higashikawa.

**Visualization:** Toshihiro Higashikawa.

**Writing – original draft:** Toshihiro Higashikawa.

**Writing – review & editing:** Toshihiro Higashikawa.

Toshihiro Higashikawa: 0000-0001-8690-5473.

Toshihiro Higashikawa orcid: 0000-0001-8690-5473.
